# Clinical encounter with three cancer patients affected by groundwater contamination at Camp Lejeune: a case series and review of the literature

**DOI:** 10.1186/s13256-022-03501-9

**Published:** 2022-07-12

**Authors:** Kyungsuk Jung, Aziz Khan, Robert Mocharnuk, Susan Olivo-Marston, Justin T. McDaniel

**Affiliations:** 1 Department of Hematology/Oncology, School of Medicine, Southern Illinois University, 315 W Carpenter St Clinic B, Springfield, IL 62702 USA; 2 Department of Microbiology, Immunology and Cell Biology, School of Medicine, Southern Illinois University, Springfield, IL USA; 3 School of Human Sciences, Southern Illinois University, Carbondale, IL USA

**Keywords:** Perchloroethylene (PCE), Trichloroethylene (TCE), Benzene, Chemical carcinogen

## Abstract

**Background:**

Advanced understanding of tumor biology has recently revealed the complexity of cancer genetics, intra/inter-tumor heterogeneity, and diverse mechanisms of resistance to cancer treatment. In turn, there has been a growing interest in cancer prevention and minimizing exposure to potential environmental carcinogens that surround us. In the 1980s, several chemical carcinogens, including perchloroethylene (PCE), trichloroethylene (TCE), and benzene, were detected in water systems supplying Camp Lejeune, a US Marine Corps Base Camp located in North Carolina.

**Case presentation:**

This article presents three cases of cancer patients who have lived at Camp Lejeune, and, decades later, came to our clinic located 1000 miles from the original exposure site. The first patient is a young Caucasian man who was diagnosed with T cell acute lymphoblastic leukemia at the age of 37, and the second patient is a Caucasian man who had multiple types of cancer in the prostate, lung, and colon as well as chronic lymphocytic leukemia in his 60s and 70s. The third patient is another Caucasian man who had recurrent skin cancers of different histology, namely basal cell carcinomas, squamous cell carcinomas, and melanoma, from his 50s to 70s.

**Conclusions:**

The US Congress passed the Honoring America’s Veterans and Caring for Camp Lejeune Families Act in 2012, which covers appropriate medical care for the people affected by the contamination. We hope that this article raises awareness about the history of Camp Lejeune’s water contamination among cancer care providers, so the affected patients can receive appropriate medical coverage and cancer screening across the country.

**Supplementary Information:**

The online version contains supplementary material available at https://doi.org/10.1186/s13256-022-03501-9.

## Background

Camp Lejeune is a US Marine Corps Base Camp covering 256 square miles on the Atlantic Seaboard in Onslow County, North Carolina. Since its establishment in 1942, it has provided training facilities and barracks for recruits as well as housing units for enlisted personnel and their families. The camp also contains nonresidential facilities such as administrative offices, hospitals, schools, day-care centers, and recreational sites. It is estimated that approximately 170,000 active-duty personnel, family members, and civilian employees live in or around the camp at any given time [1].

**Fig. 1:**
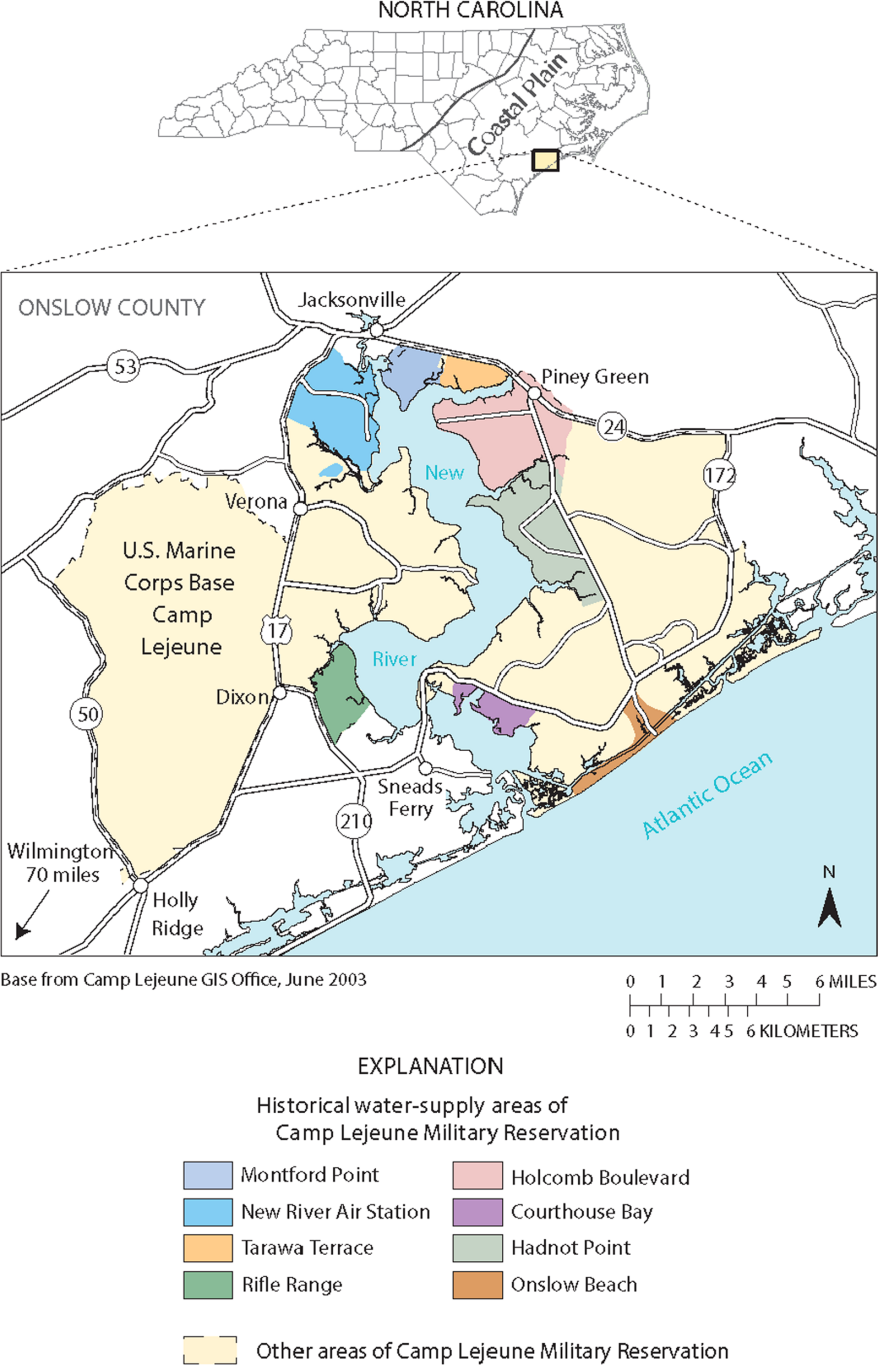
Map of Camp Lejeune and the water distribution areas. *Source* Maslia ML. Expert Panel Assessing ATSDR’s Methods and Analyses for Historical Reconstruction of Groundwater Resources and Distribution of Drinking Water at Hadnot Point, Holcomb Boulevard, and Vicinity, U.S. Marine Corps Base Camp Lejeune, North Carolina. Prepared by Eastern Research Group, Inc., Atlanta, GA. Prepared for Agency for Toxic Substances and Disease Registry (ATSDR), Atlanta, GA. April 29–30, 2009; p. 149.

In August 1982, a routine water sample screening at one of the water treatment plants in the camp detected high levels of halogenated hydrocarbons [2]. Subsequent analysis confirmed that these included perchloroethylene (PCE), trichloroethylene (TCE), and their degradation products—dichloroethylene (DCE) and vinyl chloride. The levels of these compounds were well above the maximum contaminant levels (MCL) of 5 parts per billion (ppb; µg/L) which is determined by the US Environmental Protection Agency (EPA). Further investigation in 1985 revealed that several water-supply wells which collected groundwater were the sources of contamination. It was postulated that the contaminants leached into the water-supply wells from underground water tables, and were subsequently pumped into the water treatment plants where the water was mixed and eventually distributed to both residential and nonresidential portions of the camp. The contaminated wells supplied water to Tarawa Terrace and Hadnot Point, but the Hadnot Point water system was also responsible for distributing water to housing on Holcomb Boulevard until 1972, and then intermittently thereafter (Fig. 1). The contaminated water-supply wells were eventually shut down from February to April 1985.

In April 1985, the North Carolina Department of Natural Resources and Community Development (NCDNRCD) analyzed water samples taken from the affected water-supply wells plus the monitoring wells installed for the remedial investigation. This information, combined with underground water table data that indicated a southeast direction of water flow, narrowed the contamination source to an off-base dry-cleaner [3]. The dry-cleaning establishment was geographically located on the northwest edge of the PCE plume and in close proximity to several of the water-supply wells servicing Tarawa Terrace. The owner of the business stated in a deposition that the cleaner had routinely used PCE for dry-cleaning since it opened its business in 1953 [4]. He indicated that PCE was used as a solvent for clothes as they were spun in the wheel of the dry-cleaning machine. He also stated that the waste product was gathered in powder form and was used to fill potholes. This contamination scenario was corroborated by an extremely high level of PCE from a monitoring well that was installed close to the dry-cleaning facility [5].

Other volatile organic compounds (VOC) were also potentially involved. Personnel from the US Geological Survey (USGS) reported a “strong gasoline odor” at one of the water-supply wells during a routine reconnaissance in October 1986 [5]. A review of records traced it back to various gasoline leaks and spills from underground storage tanks located near the Tarawa Terrace shopping center. Several buildings with underground storage tanks were identified in the vicinity that served as gasoline stations [2]. Of note, there was an accidental discharge of 4400 gallons of unleaded gasoline to the subsurface on September 21, 1985, and leakage of a 3000-gallon tank of leaded gasoline on July 23, 1986 [2]. In addition, a more comprehensive assessment of the local contamination and toxic release history suggested that small leaks of gas probably started in the 1950s [5]. As of May 4, 1987, it was estimated that there was more than 2 feet of floating gasoline above the water table around the Tarawa Terrace shopping center [6]. Compared to Tarawa Terrace, the water-supply contamination scenario for Hadnot Point was more complex. There were multiple potential sources of pollutants surrounding the Hadnot Point supply wells, including hazardous waste sites, fire training areas, and industrial storage lots. Several pieces of evidence suggested that the magnitude of groundwater contamination was much more extensive at Hadnot Point than at Tarawa Terrace [5].

Currently, most of the marines and their families who once lived at Camp Lejeune during the affected period have moved to different areas of the United States, and they now carry a high risk of developing cancers that are potentially associated with the abovementioned chemical carcinogens. In this article, we present the cases of three patients who lived at Camp Lejeune and developed a rare type or multiple different types of cancers decades later. Then, we review epidemiological studies that examined associations between the chemicals found in the Camp Lejeune water supply and specific cancer types. We hope that this report will raise awareness of water contamination as an important source of carcinogenesis among cancer care providers as well as the general public.

## Case presentation

### Patient #1

The first patient is a 37-year-old Caucasian man who had been relatively healthy until he presented with severe fatigue and muscle ache that persisted for several months. He visited his primary care doctor, and a complete blood count (CBC) revealed a high leukocyte count and the presence of peripheral blasts. When he was sent to an emergency room, his total white blood cell (WBC) count was recorded at 20.5 k/mm^3^, hemoglobin was 11.6 g/dL, and the platelet count was 146 k/mm^3^. He underwent a bone marrow biopsy that revealed hypercellular marrow with involvement of T lymphoblastic leukemia. Cytogenetic and molecular analysis was positive for del 11q23 (*KMT2A*), and rearrangement of 14q32 (*IGH*). BCR-ABL minor fusion transcript (p190) was detected in a qualitative test, but it was not detected in a quantitative test. The discrepancy was due to a very low level of BCR-ABL fusion in the specimen. Hence the mutation was unlikely a pathogenic driver. Fortunately, there was no evidence of central nervous system (CNS) involvement.

His father was a marine, and their family moved to Camp Lejeune in April 1985. The patient was 20 months old when they moved there, and they lived in Tarawa Terrace until 1992. When he grew up, he served in the Marine Corps himself from 2003 to 2008. After discharge, the patient had different jobs, but he had no known occupational exposure to hazardous chemicals. The patient and his family were made aware of the water contamination problem at Camp Lejeune when they received a letter from the US Navy in 2012. He never smoked and does not drink alcohol. He developed anxiety after the diagnosis of leukemia but had no other comorbidities. There is no family history of malignancy to his knowledge. He has one brother, who was *in utero* when the family moved to Camp Lejeune, and his brother has not been diagnosed with any cancer so far.

The patient was started on the Linker regimen for T cell acute lymphoblastic leukemia (ALL). Following completion of induction chemotherapy (cycle 1A), a bone marrow biopsy was normocellular, with mature tri-lineage hematopoiesis. Repeat next-generation sequencing (NGS) was negative for any genetic mutation, indicating no measurable residual disease (MRD). The patient received cycles 1B, 1C, 2A, and 2B of the Linker regimen, and then underwent an allogeneic stem cell transplant. He tolerated the procedure well, and his leukemia remains in complete remission.

### Patient #2

The second patient is a Caucasian male who has been diagnosed with multiple different types of cancer. He was first diagnosed with prostate adenocarcinoma at the age of 68 after presenting with elevated prostate-specific antigen (PSA) of 4.5 ng/mL. An ultrasound-guided biopsy of a 1 cm nodule in the left lobe was positive for clinical-stage T2aN0 prostate adenocarcinoma. The Gleason score was 3 + 4, and the tumor involved 10% of the biopsy specimen. He underwent brachytherapy with the implantation of iodine-125 seeds. And, at age 74, he was diagnosed with colon adenocarcinoma. A large ascending colon mass was found on a computed tomography (CT) scan during a workup for unexplained anemia. Subsequently, he underwent a right hemicolectomy for a high-grade, poorly differentiated pT4bN0 tumor. After surgery, he received adjuvant FOLFOX chemotherapy and has remained colon cancer-free per radiographic imaging and endoscopic surveillance. He was also diagnosed with lung cancer at the age of 75. A series of CT scans detected a slowly but progressively enlarging mass in the right upper lobe of the lung. When it was brought up as a worrisome finding, the size of the tumor was 10 × 13 mm. He declined to undergo biopsy due to the high risks of the procedure, opting instead for a positron emission tomography (PET) scan. This study confirmed the presence of the right upper lobe pulmonary nodule with a measured standardized uptake value (SUV) of 8.8. After an interdisciplinary discussion, the consensus decision was to obliterate the mass with stereotactic body radiation therapy (SBRT). The patient received a protracted course of 12 fractions of radiation at ×400 cGy per dose, and there has been no evidence of recurrent disease since then.

At the age of 78, he was diagnosed with chronic lymphocytic leukemia (CLL). He had intermittent thrombocytopenia for several years, but workup for the lymphoproliferative disease was negative. However, a recent bone marrow biopsy was positive for kappa monotypic B cells, consistent with CLL. Then, the patient developed Evans syndrome with progressive thrombocytopenia and hemolytic anemia. He was initially treated with rituximab plus prednisone. However, without improvement in the cell counts, the treatment was subsequently switched to a single-agent ibrutinib. After the switch of therapy, his blood counts have stabilized.

This patient was stationed at Camp Lejeune from 1962 to 1987 as an enlisted marine. He lived in a trailer park in Camp Johnson, formerly named Camp Knox. He has been an occasional pipe smoker for about 20 years but had no other exposure to toxic chemicals except for the contaminated water supply. He does not drink alcohol. Although an association is less clear, he and his wife had difficulty conceiving additional children for 10 years after the birth of their first child. His other comorbidities include coronary artery disease requiring stent placement, type 2 diabetes, and depression. There is no one else in the family who had cancer as far as he knows.

### Patient #3

The third patient is a 77-year-old Caucasian man who has a history of recurrent nasal papilloma and multiple skin cancers. He developed a severe *de novo* allergy while living at Camp Lejeune. Since then, he has undergone multiple surgical resections for recurrent inverted nasal papilloma. In addition, he has received nitrous oxide cryotherapy and repeated surgical resections for multiple and recurrent basal cell carcinomas and squamous cell carcinomas of the skin. Most recently, at the age of 77, he was diagnosed with melanoma of the right supraclavicular chest for which he underwent wide local excision with clear margins.

He lived at Camp Lejeune while serving as an active-duty marine for 2 years from 1967 to 1968. During the first year, he lived in a barrack in the division headquarters, located at Hadnot Point. During his second year, he and his wife moved to a married housing unit in Tarawa Terrace. Following his discharge from the service, he worked in a suture and contact lens manufacturing factory at Johnson & Johnson, but he denied any occupational exposure to chemicals or toxins. He is a never-smoker and drinks alcohol occasionally. He has a medical history of atrial fibrillation, coronary artery disease, obstructive sleep apnea, and interstitial pulmonary fibrosis. His mother, who was a heavy smoker, died of lung cancer, but no one else in his family has had cancer. Timelines of events for each patient are summarized in Table 1.

**Table 1:** Timeline of events

Time	Patient #1
1985–1992	Lived at Tarawa Terrace
Dec 2020	Diagnosed with acute lymphoblastic leukemia
	Started Linker regimen—course 1 (induction)
	Achieved complete remission in bone marrow biopsy
Jan–Apr 2021	Linker regimen—courses 2, 3, 4, 5 (consolidation)
May 2021	Allogeneic stem cell transplant
Oct 2021	Last follow-up

Time	Patient #2
1962–1987	Lived at Camp Johnson (formerly called Camp Knox)
Feb 2011	Diagnosed with prostate adenocarcinoma
Apr 2011	Brachytherapy for prostate cancer
Jun 2016	Developed autoimmune hemolytic anemia with a monoclonal B cell lymphocytosis
	Treated with steroid and rituximab
Sep 2016	Bone marrow biopsy did not reveal any lymphoproliferative disorder
Oct 2016	Diagnosed with colon adenocarcinoma
	Underwent a right hemicolectomy
	CT of the chest revealed a 6 mm speculated nodule in the right upper lobe of the lung
Dec 2016–May 2017	Adjuvant chemotherapy (FOLFOX) for colon cancer
Dec 2017	The lung mass increased in size to 1.2 cm in the chest CT
May 2018	The size of the lung mass increased to 1.3 cm with FDG uptake similar to that of the blood pool
May–Jun 2018	Stereotactic body radiation therapy (SBRT) for the lung mass
Sep 2020	Bone marrow biopsy which was performed for persistent thrombocytopenia revealed kappa monotypic B cell lymphocytosis
Sep 2021	Started ibrutinib for CLL
May 2022	Last follow-up

Time	Patient #3
1967–1968	Lived at Hadnot Point and Tarawa Terrace
2002–2020	Recurrent basal cell carcinoma and squamous cell carcinoma in different areas of skin
Dec 2020	Diagnosed with melanoma in the right upper chest
Nov 2020	Wide local excision of skin with no residual disease (melanoma *in situ*)
Mar 2021	Resection of ethmoid inverted papilloma
Jan 2022	Last follow-up

*CLL* chronic lymphocytic leukemia, *CT* computed tomography, *FDG*, fluorodeoxyglucose

## A review of epidemiological studies

Sir Bradford Hill proposed nine criteria for determining an association between a cause and a disease.[7] Realistically, the assessment of potential carcinogens is heavily reliant upon epidemiological data due to a long latency period from the initial exposure to the development of cancer. Moreover, some cancers occur at a relatively low frequency, requiring long-term monitoring of a large cohort. Nonetheless, there have been several case–control and ecological studies that have suggested an association between the contaminants found in the Camp Lejeune water supply and several different cancers. However, these results must be interpreted cautiously, as there are inherent limitations in the retrospective analyses of such large datasets. Among the studies presented here, the strength of association may vary for the same chemical because different assumptions were made when calculating group exposures based on histological data. The possibility of co-contaminants confounding the health of affected individuals also cannot be ruled out. Being mindful of these unavoidable limitations, we review the following analyses of large patient cohorts that have been thoroughly studied using rigorous standards.

### PCE and TCE

**Fig. 2:**
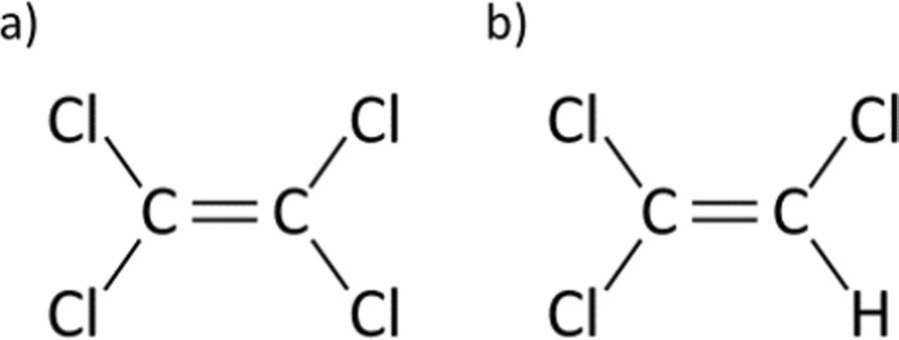
Chemical structures of **a** PCE and **b** TCE

PCE (also known as “perc”) and TCE are both halogenated solvents widely used for industrial purposes (Fig. 2). They are colorless, noninflammable, and volatile liquids with an ether-like odor. The vapor from PCE and TCE is heavy, and it readily contaminates ambient urban air. These chemicals are used for dry-cleaning, textile processing, and metal degreasing, and as feedstock in the production of chlorinated chemicals [8, 9]. PCE is relatively stable in its natural state, but it decomposes slowly in contact with moisture. Thus, PCE, TCE, and their by-products, DCE and hydrogen chloride, are commonly found as co-contaminants. These molecules can be absorbed by humans via inhalation or ingestion of contaminated vapor or water, whereas dermal absorption is considered a minor route [10]. PCE and TCE have been classified as probable and known human carcinogens, respectively, by the US EPA, and thus the goal is to achieve zero public exposure. However, the MCL was set at 5 ppb (µg/L) for both PCE and TCE because this is the lowest concentration that is reliably detectable [11]. The Agency for Toxic Substances and Disease Registry (ATSDR) has developed the Hazardous Substance Release and Health Effects Database (HazDat) to collect public health data and compile lists of contaminants specific to known hazardous waste sites. In this database, the most frequently found single-agent contaminant in groundwater was TCE (42.4%), and the most frequently found combination of substances was TCE and PCE (23.5%) [12].

In 1976 and 1978, high levels of PCE were detected in two different areas of Rhode Island by the US EPA, and vinyl liners inside the water distribution pipe were identified as the source of contamination. The vinyl-lined pipes were installed in the 1960s to treat water acidity, and the inner surface of the pipe was coated with vinyl toluene resin in which the solvent used was PCE [13]. This discovery prompted an inspection of water pipes in other states of New England, and an investigation by Massachusetts revealed that a considerable length of the problematic pipes was installed in the upper Cape Cod area, including Barnstable, Bourne, Falmouth, Mashpee, and Sandwich. In certain sites in Falmouth, where water flow was low, the level of PCE reached as high as 18,000 ppb [14]. By the early 1980s, either the affected taps were closed or the pipes were flushed to reduce the PCE concentrations below 40 ppb as a remedial action. Several years after the contamination was first identified, a significantly higher incidence of cancers was observed in towns located in upper Cape Cod relative to the entire state, which occurred in breast, colorectal, lung, and hematopoietic organs [15]. Several case–control studies were subsequently published. One study by Paulu *et al.* calculated estimated doses of exposure for the involved population based on the residence history, water flow characteristics, pipe age, and dimensions. For people whose exposure level was over the 90th percentile, the adjusted odds ratio (OR) for lung cancer (adjusted for sex, age, other chemical exposures, smoking status, and comorbidities) was elevated at 6.2 [95% confidence interval (CI) 1.1–31.6] after a 7-year latency period, and at 19.3 (95% CI 2.6–141.7) after a 9-year latency period [16]. Another study by Aschengrau *et al.* observed an elevated adjusted OR for leukemia among those whose exposure level was over the 90th percentile (OR 8.33, 95% CI 1.53–45.29 without latency; OR 5.84, 95% CI 1.37–24.91 with latency) [17].

Another incidence of water contamination with PCE and TCE was reported in 2002 in the city of Redlands in San Bernardino County, California. Water sample tests and hydrogeological studies from this area indicated that the PCE contamination in the water-supply wells began as early as 1980, with levels ranging from 5 to 98 ppb. TCE contamination likely occurred a decade earlier, with levels ranging between 0.09 and 97 ppb [18]. In 1991, the affected wells were removed from the water system, and the water was treated to reduce the concentrations of PCE and TCE. One ecological study calculated standardized incidence ratios (SIRs) of different cancer types in the affected area from 1988 to 1998, based on the actual incidence compared with the expected number of cancers. This study found that the incidence of melanoma and uterine cancer was significantly elevated in the Redlands area during those 10 years, with lower ends of a 99% CI above 1.0 (SIR 1.42, 99% CI 1.13–1.77 for melanoma; SIR 1.35, 99% CI 1.06–1.70 for uterine cancer). However, the prudent authors could not rule out possible confounding effects and stated that distinct area-related socioeconomic status could have impacted their level of healthcare access and vigilance to health-related issues [18].

In New Jersey, routine semi-annual water testing for VOCs became mandatory in 1984. Test results in 1984 and 1985 revealed detectable levels of non-trihalomethane (THM) VOCs, namely PCE, TCE, DCE, and 1,1,1-trichloroethane (TCA), in part of the water systems supplying 20% of the state’s population. An ecological study performed in 1990 in the potentially affected area reported an elevated SIR for leukemia in males (SIR 1.53, 95% CI 1.02–2.21). In this analysis, however, the contaminants were measured as a group of chemicals (non-THM VOC) and not individually, and the total level was only moderately elevated between 37 and 72 ppb [19]. A cohort study in 1994 categorized different levels of TCE in New Jersey water systems based on historical monitoring data in 1978–1984 and mandatory testing results in 1984–1985 and examined differences in rates of hematological malignancies. This study confirmed that the rates of non-Hodgkin’s lymphoma and leukemia were elevated in towns where the average level of TCE was 0.1–5.0 ppb or > 5.0 ppb. However, a dose–response relationship was not so evident, likely because the exposure levels were broadly stratified into < 0.1, 0.1–5.0, and > 5.0 ppb. For patients diagnosed with acute lymphocytic leukemia, for example, the age-adjusted ratio was higher for the 0.1–5.0 ppb exposure level than it was for the > 5.0 ppb level. However, when assessing all types of leukemia, there was a tendency for an increased rate with higher levels of exposure. For males, the age-adjusted rate ratio (RR) was 0.85 (95% CI 0.71–1.02) for the exposure level of 0.1–5.0 ppb, which increased to 1.10 (95% CI 0.84–1.43) for the exposure level of > 5.0 ppb. For females, the association was stronger. The age-adjusted RR was 1.13 (95% CI 0.93–1.37) for the exposure level of 0.1–5.0 ppb and 1.43 (95% CI 1.07–1.90) for the exposure level of > 5.0 ppb. Assuming the validity of this positive association, females were more likely to be diagnosed with leukemia than males. This suggests that sex differences may also play a role in the pathogenesis of leukemia following exposure to these chemicals [20].

Based on the knowledge obtained from laboratory research and epidemiological data, the World Health Organization’s (WHO) International Agency for Research on Cancer (IARC) and the US EPA provide hierarchal classification systems for potential carcinogens (Additional file 1: Tables S1, S2). The IARC and the US EPA have classified PCE as a group 2A and a group B carcinogen, respectively, or “probable human carcinogen” [21, 22]. TCE was classified as a group 1 carcinogen, “carcinogenic to humans,” per IARC standards, and a group A, that is, a “known human carcinogen,” by the US EPA standards [22, 23].

### Benzene

**Fig. 3:**
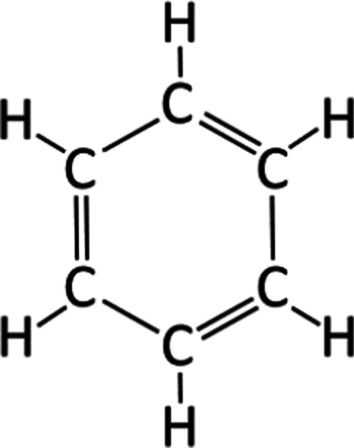
Chemical structure of benzene

Benzene is a hexagonal aromatic hydrocarbon that is volatile and highly flammable (Fig. 3). It evaporates quickly in room air and emits a unique gasoline-like odor. The vapor is heavier than air, and the liquid form floats on water. It is one of the most widely used chemicals in the United States, and functions as a solvent as well as an intermediate substrate in the various chemical production of plastics, synthetic fibers, rubbers, dyes, and drugs. Benzene is produced naturally in volcanoes and forest fires but is more commonly found in crude oil extracts and produced in the oil-refining process [8]. The general public can be exposed to low levels of benzene from automobile exhaust or industrial emissions. Cigarette smoking is also a major source of benzene exposure in the USA. Rarely, leakage from underground gasoline storage tanks or hazardous waste sites can contain benzene and contaminate drinking water, like what probably happened at Camp Lejeune [24]. Based on EPA guidelines, the MCL of benzene is 5 ppb, although the maximum contaminant level goal (MCLG) is zero [11]. In the HazDat database by ATSDR, benzene was the most frequently found air contaminant (6.0%) and the fourth most frequently found water contaminant (25.8%) in hazardous waste sites [12].

The adverse health effects of chronic benzene exposure are well known, especially hematopoietic toxicities. Animal studies have demonstrated that exposure to benzene is associated with pancytopenia and aplastic anemia as well as chromosomal changes [25, 26]. Different types of skin, gastrointestinal epithelium, liver, respiratory tract, and bone marrow tumors were observed in mice after either prolonged ingestion or inhalation of benzene [27]. The carcinogenicity of benzene was also established in human studies on exposed workers. One of the most extensively studied populations is the rubber hydrochloride workers, the so-called Pliofilm cohort. Exposure to benzene in this cohort occurred at rubber film manufacturing plants in Ohio from the 1930s to the 1970s. During the manufacturing process, the natural rubber was dissolved in benzene and was thinly spread on a conveyer belt. The benzene was then evaporated for recycling, and the rubber film was rolled and recovered from the conveyor belt. Rubber production took place at three different sites in Ohio. In one plant, workers were exposed from 1939 to 1976, and in the other two plants, exposure occurred from 1936 until 1965 [28]. Rinsky *et al.* meticulously estimated the individual risk of exposure based on company personnel records and past environmental measurements [29]. They reviewed job titles and types of work in the rubber hydrochloride plants to determine “exposure classes,” and combined these data with employment periods and the results of past industrial-hygiene measurements to calculate estimated exposure levels. Then they reviewed the vital status of the employees who worked at one of the three plants for at least 1 day between 1940 and 1965. Deaths were captured as an outcome after January 1, 1950, or earlier if the cumulative personal exposure to benzene reached at least 1 ppm-day (1 day of exposure). As a result, a total of 1165 white men were included in the cohort, contributing to 31,612 person-years at risk. Among these, 330 people died, out of which 15 people had died of lymphatic or hematopoietic cancers as of December 31, 1981. Standardized mortality ratios (SMRs) for both leukemia and multiple myeloma were significantly elevated (SMR 3.37, 95% CI 1.54–6.41 for leukemia; SMR 3.09, 95% CI 1.10–10.47 for multiple myeloma). When the cohort was divided into different exposure strata, there was a progressively increasing dose–response relationship for leukemia, but not for multiple myeloma. In an individually matched nested case–control analysis, it was confirmed that both cumulative dose and duration of exposure were higher for those diagnosed with leukemia and myeloma. Paustenbach *et al.* considered additional factors in the exposure assessment, including short-term, high-level exposures to vapors, background concentrations in the building, and dermal absorption. These additional exposures significantly increased the estimated total absorption, which was 3–5 times as high [30]. In an updated analysis that added 6 years of observation to the Pliofilm cohort, an additional five cases of leukemia were reported, and there were no additional cases of multiple myeloma. While it was suggested that a higher concentration of benzene was required for leukemogenic potential, the more general association between benzene exposure and leukemia was confirmed [31].

In China, a national occupational survey was carried out in 1981 which reported that more than 500,000 workers in 28,808 factories throughout the country were exposed to various levels of benzene between 1979 and 1981. In this report by Yin *et al.*, the investigators found a higher number of acute or chronic benzene poisoning cases among workers of shoemaking factories where benzene was mixed in the adhesive [32]. Subsequently, they monitored the cancer mortality rate among 28,460 workers who worked for at least 6 months in factories where benzene was used for painting, shoe manufacturing, rubber production, or adhesive production. Compared with workers in factories with no evidence of benzene, radiation, or other known carcinogen exposure, SMRs were elevated for leukemia, lung cancer, primary hepatocarcinoma, and stomach cancer [33]. After these reports, the Chinese Academy of Preventive Medicine (CAPM) collaborated with the US National Cancer Institute (NCI) to expand the analysis and consolidate the assessment, employing qualitative evaluation of the exposure. This analysis included 74,828 benzene-exposed workers from 1972 through 1987 in 672 factories in China and a control group of workers from workplaces where benzene was not used during the same period. The time-weighted average of benzene exposure was estimated by local industrial hygienists and occupational health personnel using measurements of historical ambient benzene levels combined with production and process information for the specific job title in each factory. This updated analysis confirmed that the incidence rate of hematological neoplasms was significantly elevated after benzene exposure. For the workers with estimated average exposure of ≥ 25, RR for all hematological neoplasms was 2.8 (95% CI 1.4–5.7). Moreover, the dose–response relationship was evident with the *p*-value for trend < 0.5, when the average exposure was divided into < 10, 10–24, and ≥ 25 [34].

Human data supporting benzene carcinogenicity after oral absorption are relatively scarce. Nonetheless, an accumulating number of studies report that water contamination by benzene does occur. For example, benzene was detected in service lines supplying drinking water to standing structures after the record California wildfire in 2018 [35]. The risk of water contamination with benzene is associated with human activities as well. Hydraulic fracturing is used at an increasing frequency for the cost-effective extraction of natural gas, but this process requires the injection of a large amount of water containing chemical additives. The risk of benzene exposure occurs when the flowback water returns to the surface, laced with the remaining additives, solvents, and petroleum-derived hydrocarbons. One study found that the benzene level increased to 148 ppb in a water sample obtained from a reservoir near coal-bed methane fracturing well sites in Sullivan County, Indiana [36]. In addition, there was a report of contaminated water on a container ship, attributed to the internal coating of the water tank [37], and there was a detectable level of benzene with other organic pollutants and organophosphorus pesticides in water reservoirs in the Haihe river basin of China [38]. In the District of Columbia, there was a cluster of leukemia-associated deaths that mysteriously occurred among the mechanics working in the Department of Public Works from 1990 to 1992. In a survey conducted to characterize exposure to carcinogens, the mechanics reported that they used petroleum to clean parts and wash their hands. They also admitted to occasionally siphoning petrol by mouth, resulting in possible oral absorption of benzene [39].

Based on the large volume of evidence characterizing benzene as a carcinogen in both animals and humans, IARC categorized benzene as a group 1 carcinogen, “carcinogenic to humans.” Under the US EPA classification, benzene is a “known carcinogen to humans,” group A. Although human data are relatively lacking for oral carcinogenicity, the US EPA has extrapolated cancer risk from the inhalation data to oral absorption [24].

## Health effects of water contamination in Camp Lejeune

After the revelation about water contamination in Camp Lejeune in 1985, multiple scientific and health investigations ensued. In 2009, the National Research Council convened a “Committee on Contaminated Drinking Water at Camp Lejeune” and released a comprehensive report on the incident. This report illustrates contamination scenarios based on groundwater models, provides exposure assessment, and reviews relevant toxicology and epidemiological studies [5]. TCE and PCE contamination was the primary interest of this report, since sampling data were available for retrospective review for these chemicals. These data repeatedly supported high levels of contamination in the supply wells and mixed water supplies. At Hadnot Point, the level of TCE in mixed water reached 1400 ppb, with a mean level of 399 ppb from 1980 to 1985. However, there was a relative lack of focus on other organic compounds, especially benzene. Benzene was detected in water samples collected at the Tarawa Terrace water treatment plant in 1985, albeit in a small amount (< 5 ppb). In the Hadnot Point water system, a significant level of benzene was detected in several supply wells, with the maximum value reaching 720 ppb in 1984. Data before 1980 were not available. It is unknown whether the high concentration of benzene in the wells reached the tap water, because the water from the supply wells was pumped to the treatment center and mixed before distribution, and the wells were cycled on and off to allow only a few wells to operate at any given time. Available records indicate that benzene has not been detected in the mixed water samples since 1980, but the data were missing in 38 out of the 52 mixed water samples from the Hadnot Point water system during the testing period from 1980 to 1985 [2]. Despite the missing data, there were reasons to suspect benzene leakage into the water supply because of multiple potential sources of pollutants in these areas, including industrial dumpsites, transformer storage lots, fire training areas, liquids disposal areas, and fuel tanks [5].

Large-scale cohort studies were conducted for the enlisted personnel and civilian employees who resided or worked at Camp Lejeune during the affected period. The first study published in 2014 by Bove *et al.* examined a cohort of 154,932 Marine and Naval personnel who were stationed at Camp Lejeune at any time between April 1975 and December 1985. The cancer mortality rate in this cohort was compared with that of a comparison cohort which consisted of 154,969 Marine and Navy personnel who were stationed in the same period at Camp Pendleton in southern California where there was no evidence of water contamination. Demographic and social/educational characteristics were comparable between the two cohorts. Hazard ratios (HRs) were calculated using Cox regression models adjusted by sex, race, rank, and education. When the base location was used as a dichotomous variable, with no regard to cumulative exposure, the HR for all-cancer mortality was elevated at 1.1 in the Camp Lejeune cohort, with a *p*-value of 0.02. However, the *p*-value was > 0.10 for any specific type of cancer mortality. Another statistical analysis that was used in this study was to include the estimated cumulative exposure as a time-varying variable. Cumulative exposure was calculated based on monthly average contaminant concentrations in the water system of each individual’s primary residence and the occupancy dates. The investigators found that higher levels of cumulative exposure to the total VOCs, which is the sum of all contaminants (TCE, PCE, trans-1,2-dichloroethylene, vinyl chloride, and benzene), were associated with elevated kidney cancer-specific mortality, and a dose–response relationship was also found in TCE and benzene with Hodgkin’s lymphoma-specific mortality [40]. For a more focused interpretation of these results, the authors created a list of cancers of primary interest, based on previous health outcomes of water contamination in Cape Cod, Massachusetts, and New Jersey. These included kidney cancer, bladder cancer, liver cancer, esophageal cancer, cervical cancer, and hematopoietic malignancies [16, 17]. In this list, the disease-specific mortality was elevated in the Camp Lejeune cohort for kidney cancer (HR 1.35, 95% CI 0.84–2.16), liver cancer (HR 1.42, 95% CI 0.92–2.20), esophageal cancer (HR 1.43, 95% CI 0.85–2.38), Hodgkin’s lymphoma (HR 1.47, 95% CI 0.71–3.06), multiple myeloma (HR 1.68, 95% CI 0.76–3.72), leukemia (HR 1.11, 95% CI 0.75–1.62), and cervical cancer (HR 1.33, 95% CI 0.24–7.32). Among those cancers with elevated HR, there was evidence of a dose-related effect in kidney cancer, cervical cancer, leukemia, and Hodgkin’s lymphoma, as they occurred primarily or exclusively in people with higher cumulative exposure [40].

A second study by the same authors looked at a different cohort affected by the contamination at Camp Lejeune. The cohort consisted of 4647 full-time civilian employees who worked between April 1973 and December 1985. All of these civilian workers resided off-base where there was no known contamination. The individuals worked in maintenance facilities, administrative offices, commissaries, and warehouses in the main area of the camp, which was served by the Hadnot Point water system. The comparison cohort at Camp Pendleton, California, included 4690 full-time civilian employees. In this analysis, the elevated mortality from all cancers was not as evident as in the previous study (HR 1.12, 95% CI 0.92–1.36). An exposure–response relationship was found with higher levels of chloride and PCE associated with increased leukemia-specific mortality. Among the diseases of primary interest, the HR for mortality was elevated for kidney cancer (HR 1.92, 95% CI 0.58–6.34), multiple myeloma (HR 1.84, 95% CI 0.43–7.58), and leukemia (HR 1.59, 95% CI 0.66–3.84). When the result was viewed together with the previous study on the cohort of Marine and Naval personnel, the cancers with elevated mortality in both studies were kidney cancer, multiple myeloma, leukemia, rectal cancer, lung cancer, and prostate cancer [41]. One potential confounder in both studies was the effect of other carcinogens. Although individual information on smoking and alcohol use was unavailable, comparing the two cohorts of similar demographics and occupations in Camp Lejeune and Camp Pendleton probably mitigated this confounding effect. The 95% CIs were wide in these studies because of the small numbers of deaths that occurred. The median age at the end of follow-up was 49 years among the Marine and Navy personnel, and 58 years among the civilians, which could be too young to sufficiently monitor cancer occurrence, let alone cancer-specific mortality.

In addition, there was a case report of hairy cell leukemia in a 55-year-old man who served at Camp Lejeune for 5 years in the 1970s [42]. Although this report does not establish a definite causal link between water contamination and hairy cell leukemia, the patient did not have any other known exposure to pesticides, petroleum, or radiation. The estimated incidence of hairy cell leukemia is three cases per one million person-year in the United States [43].

## Discussion and conclusions

Janey Ensminger, a daughter of a former Marine Corps Master Sergeant, Jerry Ensminger, was born in 1976 when her family was living at Camp Lejeune. In 1983, Janey was diagnosed with acute lymphoblastic leukemia, and in 1985, she died at the age of 9. Since then, Jerry, her father, struggled with reconciling himself to her death in light of no family history of the disease. A plausible explanation was not found until 1997 when he discovered in the local news that the tap water at Camp Lejeune was contaminated by toxic chemicals for nearly three decades. Since then, Jerry Ensminger has campaigned for the potential victims among former Camp Lejeune residents, seeking to establish a medical health registry and provide due benefits [44]. In 2012, Congress passed the Honoring America’s Veterans and Caring for Camp Lejeune Families Act, which covers medical care for veterans and family members affected by the water contamination. In response, the Institute of Medicine (IOM) published a VA clinical guidance for health conditions identified among the potential victims. To be eligible for benefits under the law, a veteran or family member must have resided at Camp Lejeune for at least 30 days between January 1, 1957, and December 31, 1987. Latency period was not considered, to maximize benefits for the veterans and families. Cancers or neoplasms that are covered under this act are esophageal cancer, lung cancer, breast cancer, bladder cancer, kidney cancer, leukemia, multiple myeloma, non-Hodgkin’s lymphoma, and myelodysplastic syndrome. Precancerous lesions are also listed, including ductal carcinoma *in situ* of the breast, Barrett’s esophagus, and monoclonal gammopathy of undetermined significance. Cancers that have been diagnosed any time during or after residency at Camp Lejeune are included, and clinically indicated screening is also covered without a co-pay for veterans and their families [45].

Initially, it seemed more than coincidental that three patients who were affected by the water contamination at Camp Lejeune were seen in one clinic located 1000 miles away. On the other hand, the encounter with these three patients speaks to the magnitude of the contamination and the numbers of patients spread throughout the United States and abroad. Based on our experience, it is likely that people who have lived in Camp Lejeune will be seen in oncology clinics across the country with a new cancer diagnosis. For those affected individuals, thorough history-taking can lead to a high level of vigilance and appropriate cancer screening, so that the eligible patients may receive financial coverage for the medical care that they need. We hope that this article raises awareness about the history of Camp Lejeune’s water contamination among cancer care providers and ultimately serves as a public reminder about the importance of chemical carcinogens in the surrounding environment.

## Supplementary Information

**Additional file 1: Table S1.** Classification of carcinogens by the IARC. **Table S2.** Hierarchical categories of carcinogens by EPA.

## Data Availability

All data supporting the findings of this study are available within the published article and its Additional file 1.

## Abbreviations

PCE: Perchloroethylene
TCE: Trichloroethylene
DCE: Dichloroethylene
MCL: Maximum contaminant level
EPA: Environmental Protection Agency
NCDNRCD: North Carolina Department of Natural Resources and Community Development
VOC: Volatile organic compound
USGS: US Geological Survey
CBC: Complete blood count
WBC: White blood cell
CNS: Central nervous system
ALL: Acute lymphoblastic leukemia
NGS: Next-generation sequencing
MRD: Measurable residual disease
PSA: Prostate-specific antigen
SBRT: Stereotactic body radiation therapy
CLL: Chronic lymphocytic leukemia
ATSDR: Agency for Toxic Substances and Disease Registry
HazDat: Hazardous Substance Release and Health Effects Database
OR: Odds ratio
CI: Confidence interval
SIR: Standardized incidence ratio
THM: Trihalomethanes
TCA: Trichloroethane
RR: Rate ratio
WHO: World Health Organization
IARC: International Agency for Research on Cancer
MCLG: Maximum contaminant level goal
SMR: Standardized mortality ratio
NCI: National Cancer Institute
HR: Hazard ratio
IOM: Institute of Medicine
