# Supplementary material for: Clinical encounter with three cancer patients affected by groundwater contamination at Camp Lejeune: a case series and review of the literature
Source: J Med Case Rep. 2022 Jul 12;16:272. doi: 10.1186/s13256-022-03501-9 (PMC9275133; doi:10.1186/s13256-022-03501-9)
Supplement: Supplementary file 1 — Additional file 1: Table S1. Classification of carcinogens by the IARC. Table S2. Hierarchical categories of carcinogens by EPA. [file 13256_2022_3501_MOESM1_ESM.docx]

Table S1. Classification of carcinogens by the IARC[1]

| Classification | | Level of evidence | | |
| --- | --- | --- | --- | --- |
|  |  | Evidence in humans | Evidence in experimental animals | Mechanistic evidence |
| Group 1 | Carcinogenic to humans | Sufficient | Not necessary | Not necessary |
|  |  | Limited or Inadequate | Sufficient | Strong (b1) |
| Group 2A | Probably carcinogenic to humans | Limited | Sufficient | Strong (b2-3), Limited, or Inadequate |
|  |  | Inadequate | Sufficient | Strong (b2) |
|  |  | Limited | Less than Sufficient | Strong (b1-3) |
|  |  | Limited or Inadequate | Not necessary | Strong (a) |
| Group 2B | Possibly carcinogenic to humans | Limited | Less than Sufficient | Limited or Inadequate |
|  |  | Inadequate | Sufficient | Strong (b3), Limited, or Inadequate |
|  |  | Inadequate | Less than Sufficient | Strong (b1-3) |
|  |  | Limited | Sufficient | Strong (c) |
| Group 3 | Not classifiable as to its carcinogenicity to humans | Inadequate | Sufficient | Strong (c) |
|  |  | All other situations not listed above | | |

1. The agent belongs to a class for which one or more members have been classified as carcinogenic or probably carcinogenic to humans
2. The agent exhibits key characteristics of carcinogens. (b1: Exposed humans, b2: Human cells or tissues, b3: Experimental systems)
3. The mechanism of carcinogenicity in animals does not operate in humans

Table S2. Hierarchical categories of carcinogens by EPA[2]

| Group A | Carcinogenic to Humans | Adequate human data to demonstrate the causal association | |
| --- | --- | --- | --- |
| Group B | Probably Carcinogenic to Humans | B1 | Sufficient evidence from animal data, but either limited human evidence(possible causal relationship, but not exclusive of alternative explanation) |
|  |  | B2 | Sufficient animal data, but little or no human data |
| Group C | Possibly Carcinogenic to Humans | Limited animal evidence and little or no human data | |
| Group D | Not Classifiable as to Human Carcinogenicity | Inadequate data either to support or refute human carcinogenicity | |
| Group E | Evidence of Non-carcinogenicity for Humans | No evidence of carcinogenicity in at least two adequate animal tests in different species or in both adequate epidemiologic and animal studies | |

**References**

1. International Agency for Research on Cancer. IARC Monographs on the Identification of Carcinogenic Hazards to Humans. World Health Organization. https://monographs.iarc.who.int/wp-content/uploads/2019/07/Preamble-2019.pdf. Accessed October 31, 2021

2. Risk Assessment Forum. Guidelines for Carcinogen Risk Assessment. U.S. Environmental Protection Agency. 1986.
